# Seasonal Variations in Antibiotic Resistance Gene Transport in the Almendares River, Havana, Cuba

**DOI:** 10.3389/fmicb.2012.00396

**Published:** 2012-11-23

**Authors:** Charles W. Knapp, Lazaro Lima, Susana Olivares-Rieumont, Emma Bowen, David Werner, David W. Graham

**Affiliations:** ^1^School of Civil Engineering and Geosciences, Newcastle UniversityNewcastle upon Tyne, UK; ^2^David Livingstone Centre for Sustainability, Department of Civil and Environmental Engineering, University of StrathclydeGlasgow, UK; ^3^Laboratorio de Análisis Ambiental, Instituto Superior de Tecnologías y Ciencias AplicadasCuidad Habana, Cuba

**Keywords:** water quality, antibiotic resistance, beta lactamase, tetracycline

## Abstract

Numerous studies have quantified antibiotic resistance genes (ARG) in rivers and streams around the world, and significant relationships have been shown that relate different pollutant outputs and increased local ARG levels. However, most studies have not considered ambient flow conditions, which can vary dramatically especially in tropical countries. Here, ARG were quantified in water column and sediment samples during the dry- and wet-seasons to assess how seasonal and other factors influence ARG transport down the Almendares River (Havana, Cuba). Eight locations were sampled and stream flow estimated during both seasons; qPCR was used to quantify four tetracycline, two erythromycin, and three beta-lactam resistance genes. ARG concentrations were higher in wet-season versus dry-season samples, which combined with higher flows, indicated much greater ARG transport downstream during the wet-season. However, water column ARG levels were more spatially variable in the dry-season than the wet-season, with the proximity of waste outfalls strongly influencing local ARG levels. Results confirm that dry-season sampling provides a useful picture of the impact of individual waste inputs on local stream ARG levels, whereas the majority of ARGs in this tropical river were transported downstream during the wet-season, possibly due to re-entrainment of ARG from sediments.

## Introduction

Growing evidence suggests that antibiotic resistance (AR) and antibiotic resistance genes (ARG) are increasing in the environment from domestic and industrial wastewater discharges (Iwane et al., 2001; Schwartz et al., 2003; Pruden et al., 2006; Li et al., 2009; Zhang et al., 2009b), agricultural use and releases (Chee-Sanford et al., 2001; Smith et al., 2004; Pei et al., 2006; Peak et al., 2007; Martinez, 2008), and other causes (Seveno et al., 2002; Knapp et al., 2010, 2011). Further, our understanding of factors that impact local ARG concentrations (as evidence of AR) is also improving. However, limited attention has been placed on rates of ARG movement in the environment, which may be of greater importance from an exposure perspective. Knowing rates of ARG mass transport is particularly important for streams and rivers, which are major receptacles of AR-affected wastes and facilitate the bulk movement of ARG away from original sources and causes, promoting ARG dissemination at grander scales (Alonso et al., 2001; Baker-Austin et al., 2006; Baquero et al., 2008; Martinez, 2008; Graham et al., 2011; Knapp et al., 2011).

Many streams display dramatic seasonal variations in terms of flow, which clearly influences bulk contaminant transport, but also influences re-entrainment of compounds that accumulate during drier periods in the sediment zone (Carvalho et al., 1999; UNEP, 2012). This may be of particular importance when considering ARG transport in tropical streams because considerable data suggest that sediments, which may be mobilized during high flow events, are primary harbors for ARG in aquatic systems (Seveno et al., 2002; Engemann et al., 2008; Zhang et al., 2009a; Allen et al., 2010). Therefore, seasonality (or extreme weather-related events) may be a critical factor when considering the scale of exposures to ARGs, especially in recreational waters where human contact is common.

Cuba, like many tropical nations, has two seasons dictated by precipitation patterns. The dry-season (rainfall <60 mm/month) occurs between November and April where rivers remain at relatively low flow levels; whereas higher flows occur in the wet-season (e.g., the hurricane season) when precipitation averages 170 mm/month between May and October (Institute of Hydraulic Resources of Cuba, 2012). As such, Cuban rivers, such as the Almendares River that passes through western Havana, can have very different flows between seasons. Such differences can result in seasonal flooding, but it also can influence contaminant transport down the river. Seasonal differences in contaminant transport (e.g., ARG) in the Almendares River may be important because the river has significant recreational activity in the estuarine zone (ACAVB, 1999), but it also has significant pollutant inputs in upstream waters.

Therefore, understanding patterns of ARG transport in the river is important to human exposures and this study was performed to assess seasonal variations in ARG transport down the river to the recreational zone. Here, we monitored sediment and water column ARG levels, flow conditions, and general water-quality conditions at eight locations in the Almendares River in wet- and dry-seasons to compare ARG levels and transport rates. Previous dry-season sampling on the Almendares River showed that tetracycline resistance determinants (*tet*^r^) were elevated in sediments downstream of metal-rich leachate inputs, whereas beta-lactam resistance genes (*bla*) were high in sediments below sewage outfalls (Graham et al., 2011). Therefore, the goal here was to determine whether similar patterns were seen in the wet-season and how seasonal differences influence actual mass transport rates of ARG down the river.

## Materials and Methods

### Field work and sample collection

**Figure 1:**
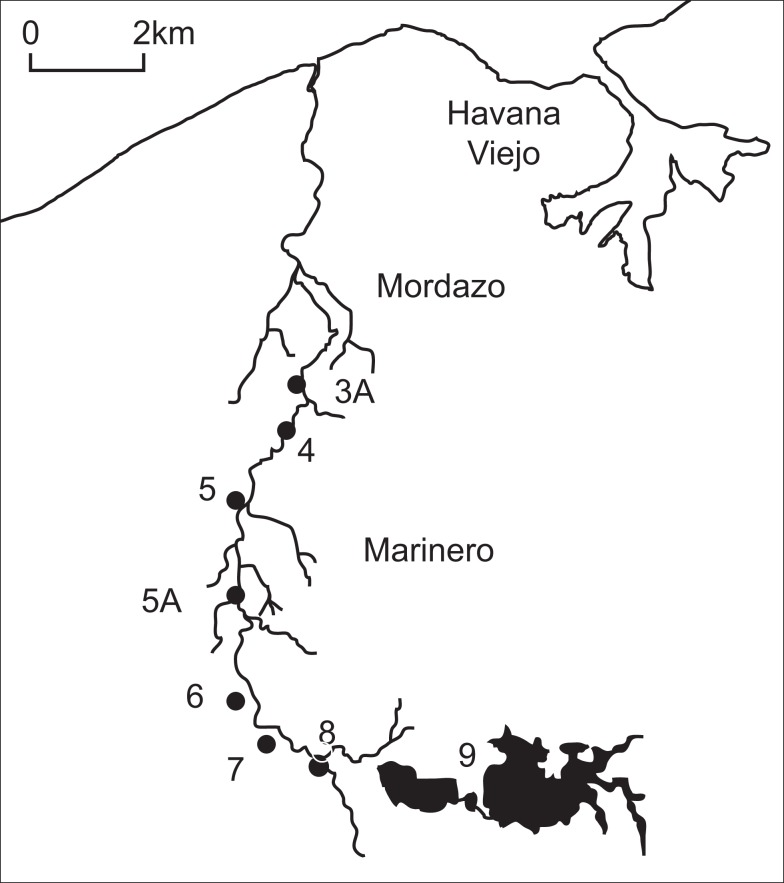
**Map of the Almendares River and sampling locations in western Havana**.

Samples of sediment and water from the overlying water column were collected from eight stations along the Almendares River during each campaign (Figure 1). The sites have been described previously (Olivares-Rieumont et al., 2005, 2007; Graham et al., 2011) and details are provided in Table 1. To make seasonal comparisons, samples were collected in April 2008 (dry-season) and the following September in the wet-season. All sampling was performed aseptically by washing instruments and gloves with 80% ethanol and flaming (when possible). Sediments and water were collected in pre-sterilized containers and transported back to the laboratory on ice. Measurements of pH, dissolved oxygen (DO), temperature, stream velocities, and river depth and width were made at each sampling station.

**Table 1:** **Description of sampling stations along the Almendares River, Cuba**.

Site	Description^a^	Dry-season flow (m^3^/s)	Wet-season flow (m^3^/s)	Depth (m)^c^	Width (m)^c^
9	Fed by an artesian spring. Shallow pool is situated in parkland on outskirts of Havana	NA	<0.16	0.42	37.4
8	Downstream of a small village. Samples were taken from pool, which was overgrown with water hyacinths	NA	0.95	1.30	5.20
7	Site parallel to a small ranch from a flowing channel with dense vegetation along the banks	0.39^b^	0.97	0.95	5.65
6	Site in municipal park above a deep pool with vegetation along banks. Often used for recreation	0.39^b^	NA	NA	NA
5A	Near the outfall from a malfunctioning waste treatment plant; an open channel with dense vegetation along banks	0.53^b^	1.93	0.42	7.45
5	Parallel to a unmanaged solid waste landfill. Channel completely overgrown with water hyacinths	0.55^b^	2.22	0.81	4.90
4	Downstream of an outlet drain from the landfill and domestic sewage discharge; some vegetation on banks	0.55^b^	2.62	0.42	6.86
3A	Site adjacent to a busy road near an open sewage outfall within a riffle zone	0.78^b^	6.66	0.82	7.00

*^a^River flows from site 9 to 3A*.

*^b^Historical data (Dominguez-Catasús et al., 2005) combined with contemporary information collected in the field*.

*^c^Data corresponding to wet-season*.

*NA, not available*.

Water samples were collected in 150 mL glass bottles. Sediment cores were obtained using ethanol-washed 1 L stainless steel cylinders pushed in to a 10 cm depth. From this bulk sediment core, 0.5 g aliquots were transferred into micro-centrifuge tubes and frozen on return to the laboratory for microbiological analysis.

### Sample processing for ARG detection

DNA were extracted within 24 h of sample collection using the UltraClean^™^ Soil DNA Isolation kit (MoBio Laboratories Inc., Carlsbad, CA, USA), employing the protocol for maximum yields. A cell disruptor was not available; therefore, cell disruption involved a combination of shaking and freeze/thaw cycles. Initially, 0.5 g of sediment samples were transferred to micro-centrifuge tubes pre-loaded with extraction buffer and glass beads. The samples were vortexed and then aggressively hand shaken for 1 min to disrupt the soil matrix. Disrupted samples were frozen at −20°C and then thawed at 70°C four times in succession to lyse cells. The remaining purification procedures followed the manufacturer’s protocol. DNA extracts were stored at −20°C prior to subsequent qPCR analysis, which was performed on all samples at the end of each sampling season.

### qPCR detection of ARG

The eluted DNA was used to quantify ARG and 16S-rRNA bacterial gene concentrations. Absolute ARG abundances were used for comparisons among sites, which are of greatest relevance to gene transport rates in the river.

All genes were quantified in duplicate by qPCR (iCycler; BioRad, Hercules, CA, USA), using carefully chosen probes and primers for specific AR determinants. The assays for tetracycline resistance [*tet*(M), *tet*(O), *tet*(Q), and *tet*(W)] and erythromycin-resistance-methylase [*erm*(B) and *erm*(E)], beta-lactam resistance (*bla*_TEM_, *bla*_SHV_, and *bla*_OXA-1_) were based on previously published methods (Knapp et al., 2010). DNA template (2 μL), appropriate primers (500 nM), and probes (200 nM, if used) were combined with iQ Supermix PCR reagent (BioRad). Reaction conditions included an initial denaturation at 95°C for 10 min, and 40–45 subsequent reaction cycles for annealing (55–60°C for 30–60 s, depending on assay), elongation and fluorescence detection (20 s at 72°C), and denaturation (20–30 s at 94°C).

All reactions were analyzed with serially diluted DNA standards of known quantity. The presence of inhibitory substances in the sample matrix was checked by spiking samples with known amounts of template and comparing differences in concentration threshold values (CT) between the matrix and controls (targeting less than one cycle difference between samples and controls). Based on pre-testing, a 1:100 dilution of extracted DNA was performed using molecular-grade water to minimize inhibitory effects of extraneous matter in the samples. PCR efficiencies (always between 75 and 110%) were determined by comparing signals from serial dilutions of samples with high DNA levels and also with plasmid controls. Correlation coefficients were >0.99 for calibration curves, and log gene abundance values were always within the linear range of detection.

### Data analysis

All data analyses were conducted using SPSS (Chicago, IL; v. 17.0) or SigmaPlot (Systat Software Inc; v. 11.0). Residual analyses were used to compare the variability between water column and sediment measured values for each season. This involved calculating the mean-square-deviations (MSD) from a linear regression. Values of ARG were log-transformed and then plotted against distance-weighted values from each station. A high MSD suggests that the measured value is more variable among stations along the river, whereas a low MSD implies spatial uniformity. MSD values for each value were then compared as a ratio between seasons (i.e., MSD_dry-season_/MSD_wet-season_) to determine whether how spatial variability differed seasonally. A ratio greater than 1.0 (i.e., the *F*-score) suggests that spatial variability is greater in the dry-season season for that value, which is important for understanding ARG exposure patterns. Principal component analysis (PCA) was based on correlation matrix with Varimax rotation.

## Results and Discussion

### Seasonal flow rates and trends in antibiotic resistance gene abundances

Flow conditions in the Almendares River varied dramatically between seasons. During the dry-season (April), estimated river flow rates ranged between 0.39 and 0.78 m^3^/s below station 8 on the river. In contrast, up to nine times higher flow rates were observed at the same stations in the wet-season (Table 1). Further, actual wet-season flow rates varied broadly among stations, ranging from nearly quiescent conditions at station 9 (< 0.16 m^3^/s) to very high flows at station 3 A (∼6.66 m^3^/s). It should be noted that flow rates in the river were particularly high during our sampling because Hurricane Ike had hit Havana about 10 days before sampling commenced. However, no additional rainfall occurred within 3 days of main sampling days. Ancillary information about environmental conditions that may affect ARG abundances, such as heavy metal and residual antibiotic concentrations, is provided in the manuscript Appendix.

**Figure 2:**
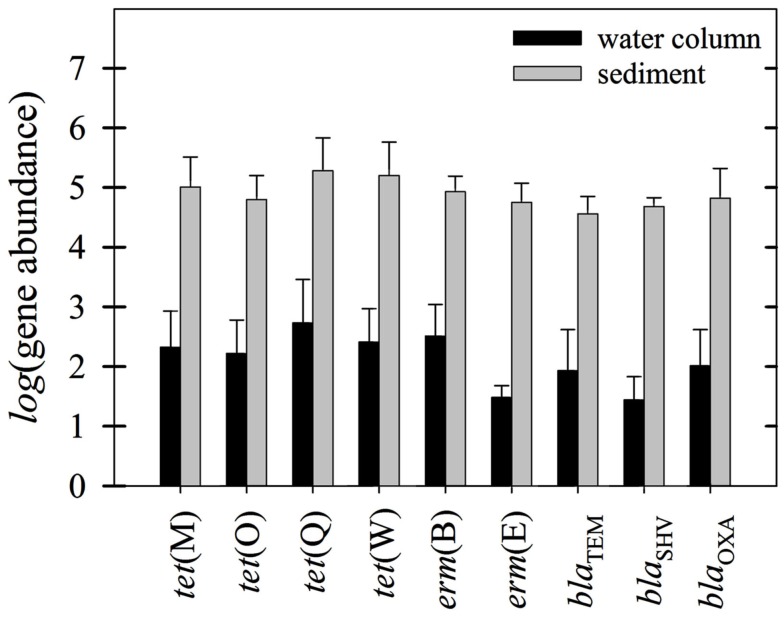
**Grand mean of all genes (absolute values; as per mL or per g-sediment) measured in the Almendares River in both seasons**. Ninety five percentage confidence intervals are denoted in parentheses.

Antibiotic resistance and 16S-rRNA gene abundances were quantified using qPCR for all water column and sediment samples from both seasons. Any comparison of ARG trends should consider: (1) relationships between water column and sediment levels, including spatial variations; (2) seasonal differences in ARG levels; and (3) differences in actual ARG mass transport downstream. In general, sediment gene concentrations were always higher than water column concentrations, typically by 2–3 orders of magnitude (Figure 2).

Given the large amount of data, three exemplar genes were chosen to facilitate detailed comparisons, which were selected based on measured water column and sediment ARG levels along the river and other factors. Specifically, *tet*(Q) and *erm*(B) were chosen because they were the highest measured ARG, whereas *bla*_TEM_ was chosen because of its epidemiological significance in Cuba (Gonzàlez Mesa et al., 2007). Additionally, PCA suggest two major components represent general ARG patterns along the river (see Appendix): a first component that includes all *tet* genes and *bla*_OXA_, and a second component that includes *bla*_SHV_, *bla*_TEM_, and *erm*(B). Therefore, *tet*(Q) and *erm*(B) were chosen because they are from different components, and *bla*_TEM_ was chosen to represent *bla* genes. As such, appropriate generalizations can be made from these select markers. However, specific comparisons also can be made for other genes using data from Tables A3 and A4 in Appendix.

### Spatial variations in water column and sediment

**Figure 3:**
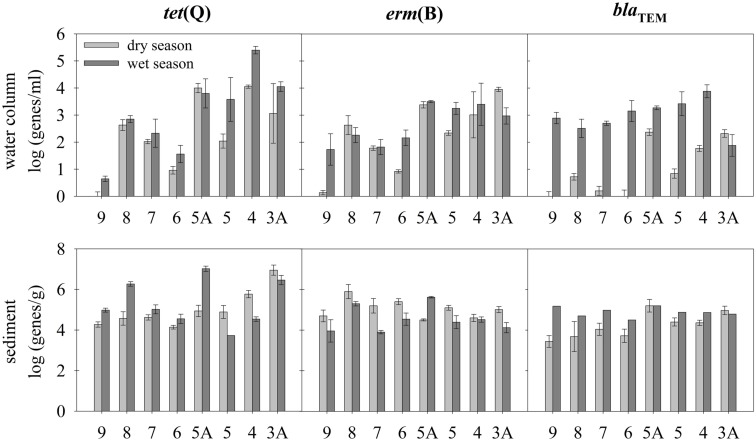
**Water column (per mL) and sediment (per g-sediment) gene abundance of exemplar genes [*tet*(Q), *bla*_TEM_, and *erm*(B)] in the Almendares River**.

There were significant variations in all gene abundances across stations in the water column and sediment for each season (ANOVA, *p* < 0.05). Absolute concentrations of *tet*(Q), *erm*(B), and *bla*_TEM_ in the water column and sediments are shown in Figure 3. Spatial and temporal patterns are complex, although general trends are apparent.

Water column ARG abundances were highly variable among stations in dry-season samples, although patterns of spatial variation for the three genes were quite similar. Spatial differences in water column gene abundances also existed in the wet-season samples, but patterns differed among the three genes, although gene abundances generally increased as one proceeded downstream.

In contrast, less acute station-to-station variability (with a few exceptions) was seen in sediment ARG levels, particularly for *erm*(B) and *bla*_TEM_. Sediment *tet*(Q) levels varied more dramatically across stations, but spatial patterns were very different between seasonal measurements. In the dry-season, sediment *tet*(Q) levels were low between stations 9 and 6, but progressively increased by three orders of magnitude by station 3A. However, no similar pattern was seen along the river in sediment samples during the wet-season, although *tet*(Q) did vary widely among stations. Further, no clear increasing trend in sediment ARG levels was apparent as one moved downstream during the wet-season, which had been seen for *tet*(Q) and *bla*_TEM_ in dry-season sediment samples.

### Seasonal differences in gene abundances and variability

In most cases, there were significant differences in gene abundances between seasons. Higher water column abundances were found during the wet-season for all genes (paired *t*-test; *p* < 0.10) except *tet*(O) and *erm*(B), which were statistically similar between seasons. In the sediments, *tet* and *erm* genes were not significantly different (paired *t*-test; *p* > 0.10), except *tet*(M) (*p* = 0.05); *bla* genes were significantly higher (*p* < 0.05) during the wet-season.

To quantify seasonal differences in spatial ARG variability, residual analysis was performed on the seasonal water column and sediment data. Specifically, MSDs were determined using linear regression of each individual ARG on the river and variances were compared for each ARG (see Table 2). Ratios of MSDs (i.e., *F* values) also were calculated to contrast differences in apparent variability between seasons. This analysis showed that water column ARG levels (with one exception) were more spatially variable in the dry-season versus the wet-season, whereas the opposite was true for sediment samples. Further, when one clumps all MSD data, significant seasonal differences in MSD become apparent; i.e., dry-season MSD are significantly greater for water column versus sediment samples (paired *t*-test; *t*_8_ = 2.84, *p* = 0.02), whereas wet-season MSD values are significantly greater in sediment than water column samples (paired *t*-test; *t*_8_ = 3.71, *p* = 0.01). This is corroborated by *F* values, which are consistently >1.0 for water column and <1.0 for sediment samples.

**Table 2:** **Interstation variability of measured sediment and water column ARG abundances during dry-season and wet-season sampling**.

	Water column			Sediment		
	Dry-season	Wet-season	*F*	Dry-season	Wet-season	*F*
*tet*(M)	0.42	0.28	1.5^a^	0.23	0.60	0.38
*tet*(O)	0.64	0.40	1.60	0.37	0.48	0.77
*tet*(Q)	0.81	0.96	0.84	0.32	1.7	0.20
*tet*(W)	1.1	0.15	7.2	0.56	0.66	0.84
*erm*(B)	0.57	0.20	2.9	0.17	1.2	0.14
*erm*(E)	0.07	0.10	0.72	0.21	0.42	0.49
*bla*_SHV_	0.23	0.16	1.5	0.10	0.60	0.16
*bla*_OXA_	0.27	0.25	1.1	0.39	0.14	2.9
*bla*_TEM_	0.64	0.08	7.7	0.33	0.44	0.75
Mean (95% CI)	0.53^b^ (0.21)	0.29 (0.18)	2.78 (1.78)	0.30 (0.09)	0.69 (0.31)	0.74 (0.56)

*Values present mean-square-deviations (MSD) of measured levels among stations along the river*.

*^a^*F* represents the ratio of dry-season versus wet-season MSD data*.

*^b^Mean MSD or *F* value among all ARG measured per season and compartment; 95% CI = 95% confidence interval*.

These observations have important practical implications. Both ARG concentrations and their spatial and compartmental variability differ broadly between seasons. This implies that one cannot use sampling from only one season to characterise ARG conditions in a river, and one also must quantify both water column and sediment ARG levels to make useful comparisons. Further, intrinsic variability from site to site demands replicate and more samples be collected to obtain a holistic picture of ARG conditions in a river, although specific locations and river compartments have value in themselves. For example, previous work showed dry-season sediment sampling was very useful for identifying key pollutant inputs to a river (Olivares-Rieumont et al., 2005, 2007; Graham et al., 2011), but we show here that neither dry-season sediment nor water column data represent conditions in the wet-season. This is particularly important when one considers ARG transport downstream in each season. If one is interested in actual mass movement of ARG downstream (not just local levels), ambient water column ARG levels are more relevant. It is well known that many sediment borne pollutants, such as heavy metals, tend to primarily migrate downstream during high flow events [e.g., UNEP (2012)], which means that differences in water column-to-sediment ARG ratio in the dry- versus wet-season must be important to ARG transport and exposure studies.

**Figure 4:**
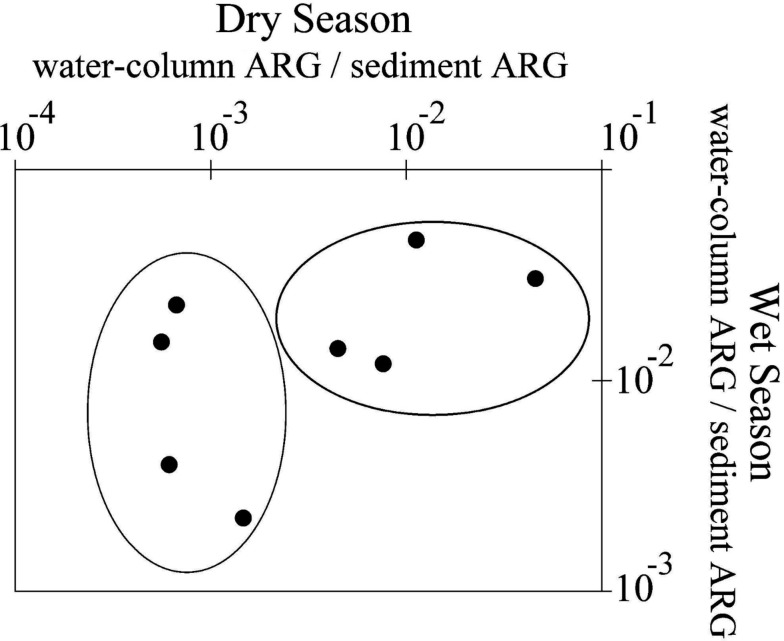
**Ratio of water column-to-sediment ARG abundances (all genes; *log*-transformed) at each station, plotted according to season**. Water column and sediment data were normalized to common sample volumes.

Therefore, water column-to-sediment ratios were calculated for all measured ARG data and ratios were plotted according to station and season, which is shown in Figure 4. The data show two distinct clusters of stations; stations 3A, 4, 5A, and 8, and stations 5, 6, 7, and 9, with dry-season ratios being an order of magnitude lower at the latter four stations relative to wet-season conditions. This is noteworthy because stations 5, 6, 7, and 9 are away from major sewage or wastes outfalls, whereas stations 3A, 4, 5A, and 8 have waste outfalls in relatively close proximity (within ∼200 m). To determine whether these patterns translate into quantifiable differences in ARG ratios for stations for each season, mean ratios for each cluster were determined and are presented in Table 3. Clearly, the local proximity of an outfall has a huge effect on water column ARG levels in the dry-season (*t*_24_ = 5.13, *p* < 0.01), whereas local outfall effects were weaker (but still significant; *t*_24_ = 3.05, *p* = 0.01) following periods of heavy rain.

**Table 3:** **Comparison of water column-to-sediment ARG ratios and ARG flux in the dry-season and wet-season for sampling near and away from major waste outfalls into the river**.

	Dry-season		Wet-season	
	Near outfalls^a^	Away from outfalls	Near outfalls	Away from outfalls
Water column-to-sediment ARG ratio^b^	0.017 (0.011)	0.0008 (0.0004)	0.025 (0.015)	0.011 (0.006)
Gene migration downstream (*log* gene abundance/s)^c^	8.37 (0.39)	7.08 (0.33)	9.49 (0.34)	8.73 (0.37)

*^a^Sampling stations near outfalls include 3A, 4, 5A, and 8, whereas stations away from outfalls include 5, 6, 7, and 9*.

*^b^Ratio of water column ARG abundances to sediment ARG abundances at the same sampling station (based on triplicates per station). Ratios were calculated for nine different ARG*.

*^c^Gene migration rates calculated based on flow rate measurements for the most downstream stations with and without a proximally close outfall, stations 3A and 5, respectively. Mean values based on flux estimates for the same nine ARG detected at both stations; 95% confidence intervals of estimates in brackets*.

This has a few key implications. First, the large variability in dry-season water column ARG levels (see Figure 3) almost certainly results from a strong local influence of outfalls. However, this influence is less important during the wet-season when river flow rates are higher. We speculate this seasonal difference partially results from higher flows that re-entrain ARG from sediments along the river (that accumulate during the dry-season), and as such, water column ARG levels become disconnected from local influences under high flow conditions. Further, it explains why water column ARG levels are more variable in the dry-season (due to greater local influence of outfalls) and sediment ARG are more variable in the wet-season (due to greater flow-related scouring and sediment transport; see Table 3).

### Downstream ARG transport in the almendares river

Among the key issues related to ARG in rivers is the actual transport of potentially medically relevant genes to locations where human exposure is most likely. Given the largest park in Havana is downstream of station 3A (Graham et al., 2011), knowing ARG transport into the park was locally important. As such, volumetric flow rates were estimated for stations 5A, 5, and 3A at the time of samplings (summarized in Table 1). Using these flow data and water column ARG levels from Figure 4, total ARG transport for the two seasons were estimated for stations close to the park: stations 3A (near outfall) and 5 (not near outfall).

Total measured ARG transport was much higher in samples collected in the wet-season (see Table 3), which was most apparent near outfalls. For example, ARG transport at station 5 was only 10^7.1^ genes/s in the dry-season, whereas ARG flux at the same location was 10^9.5^ genes/s in the wet-season. This same pattern is seen for *bla*_TEM_ genes (data not shown), which clinical data has shown is the most common resistance phenotype in *E. coli* observed in Havana hospitals (Gonzàlez Mesa et al., 2007). Therefore, our data indicate there is 90× greater movement of resistance potential (as ARG) to recreational waters in the park during the wet-season. Additionally, data from other stations suggest ARG transport is broadly higher along the whole river during the wet-season, which implies exposures are consistently higher during the wet-season, even at locations away from waste outputs.

The product of all our observations is that ARG conditions differ dramatically between the dry- and wet-season in a tropical river like the Almendares River. In the dry-season, significant spatial localization in ARG develops due to lower flow rates, increased local influence of outfalls, and reduced sediment transport. However, dry reason sampling allows one to identify major inputs that influence stream ARG. In contrast, ARG transport rate downstream is much greater in the wet-season. Further, water column ARG are more equally distributed along the river during the wet-season. This implies the potential for increased human exposure occur almost everywhere on the river during the wet-season, which is very important related to health protection guidance for recreational use. Regardless, the work here shows that seasonal sampling is critical for any river or stream system, including ambient volumetric flow measurements, which has been a major limitation in most previous studies. Therefore, we suggest this study on the Almendares River can be used to guide future studies, especially when assessing the potential for human ARG exposure is a primary goal.

## Conflict of Interest Statement

The authors declare that the research was conducted in the absence of any commercial or financial relationships that could be construed as a potential conflict of interest.

## Appendix

Extensive data were collected during the sampling endeavor along the Almendares River in 2008. Not all were presented in the manuscript. However, we present here additional information that could be used to better understand conditions along the river, and how antibiotic resistance gene (ARG) abundances and their transport could be related to the environment.

**Table A1:** **Physico-chemical conditions along the Almendares River**.

	Dry-season		Wet-season	
pH	7.30	(0.17)	8.18	(0.16)
DO (mg/L)			
(sites 6–9)	5.25	(0.98)	3.13	(3.03)
(sites 3–5A)	1.35	(0.47)	0.82	(0.75)
Temperature (°C)	28.3	(0.7)	28.4	(0.7)
Conductivity (μS/cm)	731	(48)	799	(147)
TDS (íg/L)		
(sites 6–9)	473	(49)	421	(27)
(sites 3–5A)	501	(53)	635	(126)

*Mean values are presented with 95% confidence intervals in parentheses. Dissolved oxygen (DO) and total dissolved solids (TDS) values have been segregated above and below station 5A because this station brackets a major waste outfall containing domestic and pharmaceutical wastes (Graham et al., 2011)*.

**Table A2:** **Principal component analysis of ARG abundances in sediment and water column samples**.

SEDIMENT				
**Variance explained**				
**Component**	**Eigenvalue total**		**% Variance**	**Cumulative %**

1	3.661		40.7	40.7
2	1.790		19.9	60.6

**Component matrix**				
	**Component**			
	1		2	
*tet*(M)	**0.753**		0.128	
*tet*(O)	**0.659**		0.388	
*tet*(Q)	**0.770**		0.309	
*tet*(W)	**0.858**		−0.067	
*erm*(B)	−0.043		**0.820**	
*erm*(E)	0.279		**0.410**	
*bla*_SHV_	0.003		**0.890**	
*bla*_OXA_	**0.772**		0.036	
*bla*_TEM_	0.320		**0.665**	
**WATER COLUMN**				
**Variance explained**				

**Component**	**Eigenvalue total**		**% Variance**	**Cumulative %**

1	4.124		45.8	45.8
2	1.501		16.7	62.5
3	1.250		13.9	76.4

**Component matrix**				
	**Component**			
	1	2	3	

*tet*(M)	**0.696**	−0.178	0.540	
*tet*(O)	**0.748**	0.501	0.128	
*tet*(Q)	**0.878**	−0.168	0.162	
*tet*(W)	**0.714**	0.307	0.009	
*erm*(B)	**0.831**	0.346	0.161	
*erm*(E)	0.148	**0.907**	0.027	
*bla*_SHV_	0.088	**0.742**	0.427	
*bla*_OXA_	0.167	0.223	**0.721**	
*bla*_TEM_	0.090	0.096	**0.881**	

*Calculations were based on the correlation matrix using Varimax rotation to maximize differences among components. Related parameters within each component are highlighted in bold*.

**Table A3:** **Total 16S-rNA and resistance gene abundances (per gram-sediment) in the Almendares River**.

Season/site	16S-rRNA Gram	*tet*(M) 16S	*tet*(O) 16S	*tet*(Q) 16S	*tet*(W) 16S	*erm*(B) 16S	*erm*(E) 16S	*bla*_SHV_ 16S	*bla*_OXA_ 16S	*bla*_TEM_ 16S
**DRY SEASON**										
3A	10.2 (0.3)	6.2 (0.6)	6.5 (0.9)	6.9 (0.6)	6.7 (0.5)	5.0 (0.4)	5.2 (0.7)	5.1 (0.2)	5.9 (0.1)	4.9 (0.3)
4	10.1 (0.3)	6.0 (0.2)	6.1 (0.3)	6.8 (0.3)	6.6 (0.2)	4.6 (1.0)	5.4 (0.3)	5.0 (0.5)	5.1 (1.0)	4.4 (0.3)
5	9.6 (0.4)	5.1 (0.5)	5.6 (0.5)	5.9 (0.6)	5.6 (0.6)	5.0 (0.2)	4.8 (0.4)	5.0 (0.4)	5.4 (0.8)	4.5 (0.6)
5A	9.4 (0.5)	4.1 (0.5)	4.3	4.8 (0.2)	4.7 (0.5)	4.4 (0.6)	3.7 (1.3)	4.9 (0.5)	3.9 (0.4)	5.1 (0.4)
6	9.9 (0.1)	3.8 (0.6)	3.8 (0.7)	4.1 (0.2)	bdl	5.4 (0.4)	4.0 (2.0)	5.1 (0.2)	bdl	3.8 (0.9)
7	9.6 (0.3)	4.3 (0.3)	4.2 (0.5)	4.6 (0.4)	4.3 (1.2)	5.2 (0.9)	4.7 (0.9)	4.8 (0.7)	bdl	4.0 (0.7)
8	9.3 (0.9)	4.2 (0.6)	4.3 (1.7)	4.3 (0.9)	4.4 (0.7)	5.5 (1.2)	4.2 (1.3)	4.5 (1.6)	4.1 (1.5)	3.7 (1.4)
9	9.6 (0.5)	bdl	4.3 (0.5)	4.3 (0.5)	4.3 (0.6)	4.7 (0.6)	3.8 (1.1)	4.1 (0.3)	bdl	bdl
**WET-SEASON**										
3A	10.4 (0.3)	6.1 (0.4)	5.3 (1.4)	6.5 (0.3)	6.2 (0.3)	4.1 (0.7)	5.2 (1.3)	4.2 (0.6)	5.7 (1.5)	4.8 (0.7)
4	10.4 (0.3)	5.4 (0.5)	4.8 (0.6)	4.5 (0.2)	5.5 (0.6)	4.5 (0.6)	5.3 (1.1)	4.5 (0.2)	5.6 (0.2)	4.9 (0.2)
5	10.5 (0.3)	6.3 (3.6)	4.9 (0.2)	3.5	5.1 (0.4)	4.5 (1.1)	4.8 (1.1)	4.7 (0.3)	5.8 (0.1)	4.9 (0.3)
5A	10.5 (0.4)	6.5 (0.4)	4.8 (1.4)	6.9 (0.3)	7.5 (2.9)	5.5 (0.3)	4.7 (1.2)	4.6 (0.8)	6.4 (0.3)	5.2 (0.5)
6	8.1 (0.9)	4.6 (0.7)	3.7	4.7 (1.4)	3.5	4.9 (0.0)	4.0 (1.0)	4.4 (1.6)	3.8 (1.8)	4.5 (0.7)
7	10.0 (0.3)	4.6 (1.4)	bdl	5.4 (1.6)	5.2 (0.7)	bdl	6.0 (0.1)	4.8 (0.9)	4.9	5.0 (0.5)
8	10.3 (0.2)	5.7 (0.3)	5.1 (1.3)	6.4 (0.2)	5.7 (0.8)	5.4 (0.2)	4.9 (0.4)	4.5 (0.6)	5.5 (0.8)	4.8 (0.1)
9	10.5 (0.4)	4.0 (0.7)	5.8 (6.1)	5.0 (0.3)	4.5 (0.5)	4.2 (0.8)	5.4 (0.5)	4.8 (0.6)	5.0 (0.1)	5.2 (0.3)

*All values are log-transformed and represent site-means (*n* = 5 samples/site). Ninety-five percent confidence intervals are noted in parentheses*.

**Table A4:** **Total 16S-rRNA and antibiotic resistance gene abundances (per mL water) in the water column of the Almendares River**.

Season/site	16S-rRNA Ml	*tet*(M) 16S	*tet*(O) 16S	*tet*(Q) 16S	*tet*(W) 16S	*erm*(B) 16S	*erm*(E) 16S	*bla*_SHV_ 16S	*bla*_OXA_ 16S	*bla*_TEM_ 16S
**DRY SEASON**										
3A	6.1 (0.4)	3.0 (0.3)	3.9 (0.4)	4.1 (0.4)	3.5 (0.4)	3.9 (0.4)	1.3 (0.5)	1.7 (0.4)	2.5 (0.2)	2.3 (0.2)
4	6.4 (0.5)	2.6 (0.8)	3.7 (0.1)	4.1 (0.4)	4.0 (0.5)	3.7 (0.1)	1.6 (0.9)	1.3 (0.7)	2.4 (0.6)	1.8 (0.6)
5	6.5 (0.5)	1.8 (0.4)	2.5 (0.5)	2.0 (1.3)	2.2 (0.50	2.5 (0.5)	1.3 (0.5)	0.8 (0.2)	1.7 (0.5)	0.9 (0.5)
5A	6.5 (0.2)	2.9 (1.0)	3.7 (0.4)	3.9 (0.3)	0.8 (0.8)	3.6 (0.6)	1.5 (0.3)	1.5 (0.3)	2.5 (0.3)	2.4 (0.4)
6	6.0 (0.3)	0.3 (0.3)	0.8 (0.3)	1.0 (0.2)	0.8 (0.3)	1.0 (0.2)	1.4 (0.2)	0.1 (0.5)	0.2 (0.4)	−0.6 (0.4)
7	6.1 (0.1)	1.2 (0.70	1.8 (0.2)	2.0 (0.2)	2.3 (0.1)	1.8 (0.2)	1.1 (0.5)	0.7 (0.6)	0.8 (0.3)	0.2 (0.5)
8	6.0 (0.5)	1.3 (0.7)	2.6 (0.8)	2.6 (0.2)	2.5 (0.2)	2.6 (0.8)	0.9 (0.2)	0.4 (0.6)	1.0 (0.5)	0.7 (0.6)
9	4.9 (0.1)	0.2 (0.4)	0.2 (0.1)	0.0 (0.8)	0.3 (0.1)	0.2 (0.1)	0.6 (0.6)	0.1 (0.6)	bdl	−0.5 (0.6)
**WET-SEASON**										
3A	7.6 (0.4)	3.0 (0.4)	1.0	4.0 (0.6)	3.1 (0.8)	3.0 (0.9)	1.5 (0.2)	2.0 (1.2)	1.4 (0.6)	1.9 (1.1)
4	8.5 (0.1)	4.7 (0.2)	3.5 (0.1)	5.4 (0.4)	4.1 (0.6)	3.4 (2.5)	2.1 (0.6)	2.1 (0.8)	3.0 (1.7)	3.9 (0.7)
5	8.2 (0.6)	3.8 (0.4)	2.8 (0.3)	3.6 (2.4)	3.4 (0.3)	3.3 (0.4)	2.2 (0.5)	2.5 (0.6)	2.9 (2.4)	3.5 (1.0)
5A	7.8 (0.2)	3.9 (0.3)	2.6 (0.3)	3.8 (1.4)	3.4 (0.3)	3.5 (0.2)	1.7 (0.5)	1.8 (0.6)	3.6 (0.3)	3.3 (0.3)
6	7.9 (0.4)	2.4 (0.6)	1.4 (0.6)	1.5 (0.9)	2.3 (0.3)	2.3 (1.0)	1.9 (0.5)	2.4 (0.7)	3.5 (1.3)	3.2 (1.0)
7	6.5 (0.8)	2.3 (0.8)	1.7 (0.8)	2.1 (0.5)	1.9 (0.7)	1.4 (0.6)	1.6 (0.9)	1.7 (0.8)	2.6 (1.4)	2.7 (0.8)
8	6.6 (0.3)	2.2 (0.3)	2.1 (0.9)	2.9 (0.1)	2.1 (1.2)	2.3 (0.8)	1.5 (0.4)	2.1 (0.7)	3.0 (1.1)	2.5 (1.1)
9	6.3 (0.6)	1.8 (0.9)	1.3 (1.0)	0.7 (1.9)	1.9 (0.9)	1.7 (1.3)	1.5 (1.1)	1.9 (0.6)	1.9 (1.0)	2.9 (0.5)

*All values are log-transformed and represent site-means (*n* = 5 samples/site). Ninety-five percent confidence intervals are noted in parentheses*.

**Table A5:** **Heavy metal levels in sediments along the Almendares River in μg/g**.

Station	Cadmium		Copper		Chromium		Lead		Cobalt		Zinc	
	Dry	Wet	Dry	Wet	Dry	Wet	Dry	Wet	Dry	Wet	Dry	Wet
9	0.24	2.03	11.6	57.8	95.3	128	17.3	110	8.8	24.7	46.0	88.2
	(0.02)	(0.40)	(0.6)	(7.3)	(8.3)	(6.4)	(1.5)	(26)	(0.8)	(3.0)	(2.4)	(3.0)
8	0.43	3.10	57.9	110	85.6	93.2	44.8	200	13.0	16.0	152	345
	(0.01)	(0.27)	(5.0)	(2.9)	(4.6)	(1.7)	(5.1)	(17)	(1.4)	(0.6)	(7.8)	(8)
7	0.64	1.23	90.1	63.7	139	104	59.8	69.5	14.7	19.7	186	178
	(0.05)	(0.19)	(7.4)	(3.4)	(11)	(8.2)	(5.6)	(4.7)	(1.7)	(1.2)	(15)	(11)
6	0.74	1.34	21.5	79.3	148	84.3	64.9	78.2	14.7	15.8	245	212
	(0.05)	(0.33)	(1.5)	(1.9)	(16)	(3.3)	(3.3)	(7.7)	(1.0)	(0.3)	(20)	(6)
5A	0.68	4.72	42.7	167	121	129	57.4	274	9.6	9.9	274	719
	(0.05)	(0.69)	(1.9)	(6)	(7)	(15)	(4.9)	(44)	(0.8)	(0.4)	(12)	(146)
5	0.49	2.18	137	124	120	144	31.2	155	15.5	15.7	99.4	345
	(0.04)	(0.59)	(13)	(2)	(7)	(8)	(3.0)	(29)	(1.2)	(0.8)	(11.5)	(7)
4	1.59	2.20	872	213	198	102	108	142	13.6	9.3	802	345
	(0.12)	(0.62)	(46)	(24)	(15)	(10)	(7)	(15)	(0.8)	(0.5)	(39)	(26)
3A	0.28	1.89	254	114	58.5	88.5	50.4	157	3.3	11.5	333	355
	(0.02)	(0.60)	(28)	(11)	(6.1)	(5.6)	(2.9)	(24)	(0.3)	(0.6)	(17)	(37)
Mean	0.64	2.34	186	116	124	109	54.2	148	11.6	15.3	267	323
95% CI	(0.30)	(0.78)	(200)	(36.7)	(31.1)	(15.1)	(18.6)	(46.3)	(2.88)	(3.59)	(163)	(130)

*Mean values are presented for both the dry- and wet-seasons (95% confidence intervals in parentheses)*.

*Sediment-metal levels were quantified using a Buck Scientific 210VGP Atomic Absorption Spectrometer, equipped with an air-acetylene flame and deuterium background corrector. Metals analyzed included Cd, Pb, Cu, Zn, Co, and Cr. Each sample was digested using a HF/HClO_4_/HNO_3_ mixture (1:1:2) at 200°C. Parallel analysis of certified reference SOIL 7 (IAEA) provided quality control. Detected levels in SOIL 7 were always <7% of defined values for all metals based on nine replicates*.

**Table A6:** **Antibiotic residue concentrations in the water column at sampling stations along the Almendares River in ppt**.

Station	Tetracyclines		Ampicillin		Benzyl-penicillin	
	Dry	Wet	Dry	Wet	Dry	Wet
9	10.3	15.0	279	32.9	66.0	0.0
	(28.5)	(14.6)	(238)	(26.8)	(17.5)	(0.0)
8	2.6	10.2	440	749	63.5	0.0
	(7.2)	(4.7)	(95)	(1730)	(15.0)	(0.0)
7	16.6	19.3	199	70.7	59.6	0.0
	(33.9)	(17.7)	(167)	(99.6)	(10.2)	(0.0)
6	0.0	25.0	230	74.1	60.6	0.0
	(0.0)	(15.2)	(233)	(137)	(23.3)	(0.0)
5A	155	32.2	920	653	95.9	0.0
	(130)	(14.4)	(491)	(770)	(30.4)	(0.0)
5	35.5	25.3	7590	5080	49. 6	198
	(65.5)	(14.1)	(6860)	(9510)	(27.0)	(256)
4	14.2	33.6	11400	18700	96.0	139
	(23.3)	(15.6)	(2800)	(19900)	(60.1)	(93)
3A	41.5	57.0	7930	1180	32.9	4.0
	(37.4)	(38.8)	(2660)	(1390)	(15.5)	(11.1)
Mean	34.5	27.2	3620	3310	67.8	42.6
95% CI	(35.2)	(10.0)	(3170)	(4460)	(15.3)	(54.9)

*Mean values are presented for both the dry- and wet-seasons (95% confidence intervals in parentheses)*.

*Water column antibiotic levels of three common antibiotics were quantified using the R-Biopharm Strip Reader (Darmstadt, Germany) and ELISA kits targeting tetracyclines (RIDASCREEN; R-Biopharm, Darmstadt, Germany), ampicillin (Gentaur; Kobe, Japan), and benzyl-penicillin (Gentaur; Kobe, Japan). Similar kits were not available for erythromycin or other macrolide antibiotics at the time of the study. These assays were chosen to provide “example” antibiotic levels in the river to compare patterns with observed metal and ARG data. Fifty microliters sample volumes were used in all assays (in duplicate)*.

**Table A7:** **Significant (*p* < 0.05) bivariate correlations between 16S-rRNA, ARG, sediment-metal, and water column antibiotic concentrations**.

	Dry-season		Wet-season	
	Positive correlation	Negative correlation	Positive correlation	Negative correlation
WATER COLUMN				
16S-rRNA genes	*tet*(O), ampicillin	*erm*(E)	Cu, Zn, ampicillin, benzyl-penicillin	*tet*(O), *erm*(E), *bla*_SHV_, *bla*_OXA_, *bla*_TEM_, Co
*tet*(M)	*tet*(O), *tet*(Q), *erm*(B), *bla*_SHV_, Zn	Co	*tet*(O), *tet*(Q), *tet*(W), *erm*(B), Cd, Cu, Pb, Zn	–
*tet*(O)	16S, *tet*(M), *tet*(Q), *erm*(B), *bla*_SHV_, *bla*_OXA_, *bla*_TEM_	Co	*tet*(M), *tet*(Q), *tet*(W), *erm*(B), *erm*(E), *bla*_TEM_	16S
*tet*(Q)	*tet*(M), *tet*(O), *erm*(B), *bla*_SHV_, *bla*_OXA_, *bla*_TEM_, Cu, Zn	Co	*tet*(M), *tet*(O), *tet*(W), *erm*(B), Cu	–
*tet*(W)	*erm*(B), Cd, Cu, Pb, Zn, ampicillin	–	*tet*(M), *tet*(O), *tet*(Q), *erm*(B), *erm*(E)	–
*erm*(B)	*tet*(M), *tet*(O), *tet*(Q), *tet*(W), *bla*_SHV_, bla_OXA_, *bla*_TEM_, Cu, Zn, ampicillin	Co	*tet*(M), *tet*(O), *tet*(Q), *tet*(W)	–
*erm*(E)	–	16S	*tet*(O), *tet*(W), *bla*_SHV_, *bla*_OXA_, *bla*_TEM_	16S, Cu
*bla*_SHV_	*tet*(M), *tet*(O), *tet*(Q), *erm*(B), *bla*_OXA_, *bla*_TEM_	Co	*tet*(O), *erm*(E)	16S, Cu, Zn
*bla*_OXA_	*tet*(M), *tet*(O), *tet*(Q), *erm*(B), *bla*_SHV_, *bla*_TEM_, Cu, Zn	Co	*erm*(E), *bla*_SHV_	16S
*bla*_TEM_	*tet*(M), *tet*(O), *tet*(Q), *erm*(B), *bla*_SHV_, *bla*_OXA_	Co	*tet*(O), *erm*(E), *bla*_SHV_	16S
**SEDIMENT**				
16S-rRNA genes	Cu, Zn	–	Cr, Pb	*erm*(B), *erm*(E), *bla*_SHV_, *bla*_TEM_
*tet*(M)	*tet*(O), *tet*(Q), *tet*(W), *bla*_OXA_, Cu, Zn, ampicillin	–	–	–
*tet*(O)	*tet*(M), *tet*(Q), *tet*(W), *bla*_OXA_, ampicillin	–	–	–
*tet*(Q)	*tet*(M), *tet*(O), *tet*(W), *bla*_OXA_, Cu, Zn, ampicillin	*erm*(B)	*tet*(M), *tet*(W), *erm*(B), *bla*_OXA_	Benzyl-penicillin
*tet*(W)	*tet*(M), *tet*(O), *tet*(Q), *erm*(E), *bla*_OXA_, Cu, Zn, ampicillin	–	Cd, Pb, Zn	–
*erm*(B)	–	*tet*(Q), Cu, Zn	*tet*(Q), *bla*_SHV_	16S
*erm*(E)	*tet*(W)	–	*bla*_SHV_, *bla*_TEM_	16S, Cd, Pb
*bla*_SHV_	–	–	*tet*(O), *erm*(B), *erm*(E), *bla*_TEM_	Pb
*bla*_OXA_	*tet*(M), *tet*(O), *tet*(Q), *tet*(W), ampicillin	–	*tet*(Q), Pb, Zn	Co
*bla*_TEM_	*bla*_SHV_, tetracyclines	–	*erm*(B), *erm*(E), *bla*_SHV_	–

Significance levels, *p *< 0.01

**Denote seasonal correlations that are *p *< 0.01*.
